# Fumigant Toxicity of Lamiaceae Plant Essential Oils and Blends of Their Constituents against Adult Rice Weevil *Sitophilus oryzae*

**DOI:** 10.3390/molecules21030361

**Published:** 2016-03-16

**Authors:** Sung-Woong Kim, Hyo-Rim Lee, Myeong-Jin Jang, Chan-Sik Jung, Il-Kwon Park

**Affiliations:** 1 Department of Forest Sciences, College of Agriculture and Life Sciences, Seoul National University, Seoul 08826, Korea; ksw1945@naver.com (S.-W.K.); kazu21c@naver.com (H.-R.L.); 2 Division of Forest Insect Pests and Diseases, Korea Forest Research Institute, Seoul 02455, Korea; pokey12@snu.ac.kr (M.-J.J.); csjung@korea.kr (C.-S.J.); 3 Research Institute of Agriculture and Life Science, College of Agriculture and Life Sciences, Seoul National University, Seoul 08826, Korea

**Keywords:** plant essential oils, lamiaceae, fumigant toxicity, rice weevil, artificial blends

## Abstract

To find a new and safe alternative to conventional insecticides, we evaluated the fumigant toxicity of eight Lamiaceae essential oils and their constituents against the adult rice weevil *Sitophilus oryzae*. Of the eight species tested, hyssop (*Hyssopus offcinalis*), majoram (*Origanum majorana*), and *Thymus zygis* essential oils showed strong fumigant toxicity against *S. oryzae* adults at 25 mg/L air concentration. Constituents of active essential oils were analyzed by gas chromatography coupled to flame ionization detector (FID) and gas chromatography-mass spectrometry. A total of **13**, **15**, and **17** compounds were identified from hyssop, majoram, and *Thymus zygis* essential oils, respectively. Pinocamphone and isopinocamphone were isolated by open column chromatography. Among the test compounds, pinocamphone and isopinocamphone showed the strongest fumigant toxicity against *S. oryzae*. Sabinene hydrate, linalool, α-terpineol, and terpinen-4-ol exhibited 100% fumigant toxicity against *S. oryzae* at 3.9 mg/L air concentration. The measured toxicity of the artificial blends of the constituents identified in hyssop, majoram, and *Thymus zygis* oils indicated that isopinocamphone, terpine-4-ol, and linalool were major contributors to the fumigant toxicity of the artificial blend, respectively.

## 1. Introduction

The rice weevil, *Sitophilus oryzae* (L.) is a serious insect pest of stored cereals around the world [1]. Several synthetic pesticides, including organophosphate, pyrethroids, or gaseous pesticides, have been widely used for the control of rice weevils [2]. Although these pesticides have greatly contributed to the management of the rice weevil population, they have caused several side effects including toxicity problems in humans and other animals, development of resistance, and environment pollution. Therefore, developing safe alternatives for conventional pesticides is urgently needed. Plant essential oils are good alternatives. They could be easily obtained by steam distillation and contain many bioactive compounds with fumigant toxicity against insect pests of stored cereals [1,2,3,4,5]. Another advantage of plant essential oils and their constituents is high volatility [6,7,8]. The high volatility of plant essential oils and their constituents reduces concern for their residues on stored grains.

Plants belonging to the family Lamiaceae are distributed around the world and are frequently aromatic. Many Lamiaceae plants have been used as traditional remedies for treating various ailments including coughs, digestive disorders, and heart disorders [9]. Plant essential oils of Lamiaceae have also been documented to show insecticidal and repellent activity against several insect pests [10,11,12]. Petrakis *et al.* [10] reported the effect of three Lamiaceae family members; *i.e.*, *Origanum majorana*, *Mentha pulegium*, and *Melissa offcinalis*, on longevity and fecundity of the aphid *Myzus persicae*. Ogendo *et al.* [11] evaluated the fumigant and repellent effects of *Ocimum gratissimum* essential oil and its constituents, β-(*Z*)-ocimene and eugenol, on adults of *Sitophilus oryzae*, *Tribolium castaneum*, *Oryzaephilus surinamensis*, *Rhyzopertha dominica*, and *Callosobruchus chinensis*. Rozman *et al.* [12] investigated the toxicity of the naturally occurring compounds of Lamiaceae and Lauraceae essential oils against three stored-product insect pests including *Sitophilus oryzae*, *Rhyzopertha dominica*, and *Tribolium castaneum*.

In this study, we evaluated the fumigant toxicity of eight Lamiaceae plant essential oils and their constituents against the rice weevil *S. oryzae* to find new alternatives for conventional pesticides. Furthermore, fumigant toxicities of artificial blends made by constituents of active oils were also investigated to determine the main contributor to toxicity of active oils.

## 2. Results and Discussion

### 2.1. Fumigant Toxicities of Lamiaceae Plant Essential Oils

The fumigant toxicities of 8 Lamiaceae plant essential oils against adults of rice weevil are shown in Table 1. Among the test plant essential oils, hyssop, majoram and *Thymus zygis* oils showed 100% fumigant toxicity at 25 mg/L air concentration. The fumigant toxicities of hyssop and *Thymus zygis* oils were 100% and 74% at 6.5 mg/L air concentration but were reduced to 6% and 6% at 3.0 mg/L air concentration, respectively. The fumigant toxicity of majoram was 80% at 12.5 mg/L air concentration but decreased to 14% at 6.5 mg/L air concentration. Other oils revealed moderate or weak fumigant toxicity at 25 mg/L air concentration. Insecticidal or antimicrobial activities of plant essential oils tested in the current study have been documented in previous studies [13,14,15,16,17,18]. Insecticidal activities of majoram oil and its components against the German cockroach *Blattella germanic*a were well studied by Jang *et al.* [13]. Antimicrobial and antifungal activities of *Thymus zygis* and hyssop essential oils have been documented in previous studies [14,15,16,17,18], but there has been no report on their insecticidal activity against stored product insect pests. Our results and previous studies indicated that the plant essential oils used in the current study could be good resources for managing the population of stored product insect pests. Furthermore, we report the insecticidal activities of hyssop and *Thymus zygis* essential oils for the first time.

### 2.2. Chemical Analysis of Active Plant Essential Oils

Chemical analyses of hyssop, majoram, and *Thymus zygis* essential oils are shown in Table 2. The main components of hyssop oils are isopinocamphone (35.68%), followed by pinocamphone (13.25%), camphor (13.25%), β-pinene (11.67%), linalyl acetate (1.40%), and myrcene (1.32%). Daniele *et al.* [18] analyzed the chemical components of hyssop oils, and isopinocamphone (29%), pinocamphone (18.5%), β-pinene (10.8%) and camphor (5.3%) were identified as the main compounds. A previous study and ours showed similar results, although there was a difference in the composition rate and minor components. Galambosi and Peura [19] have already reported that there is a wide difference in the composition of plant essential oils according to production conditions, such as cultivation localities, harvesting date, storage time, climate, and soil factors. A total of 15 compounds were identified in majoram essential oil. The most abundant compound was terpinen-4-ol (22.96%), followed by linalool (15.32%), γ-terpinene (12.92%), *p*-cymene (6.37%), α-terpinene (5.40%), limonene (3.22%), *α*-terpineol (2.50%), and sabinene hydrate (2.04%). The composition ratios of other compounds were less than 2%. Chemical analysis of majoram oil was reported by El-seedi *et al.* [20]. The composition ratio of terpinen-4-ol (55.6%) was the highest, followed by α-terpineol (9.5%), linalool (3.7%), γ*-*terpinene (3.6%), and p-menth-2-en-1-ol (3.0%). Linalool (40.14%) was identified as the most abundant component in *Thymus zygis* oil. Other compounds including terpinen-4-ol (8.83%), myrcene (5.88%), sabinene hydrate (5.35%), and *p*-cymene (4.14%) were identified as main components. Gonçalves *et al.* [15] investigated the antifungal and cytotoxic activity of *Thymus zygis* oils and its components. Linaool (30.0%), 1,8-cineole (12.0%), *p*-cymene (11.0%), thymol (8.0%), camphene (3.9%), and γ-terpinene (3.8%) were determined to be the main components.

### 2.3. Fumigant Toxicities of Constituents Identified from Active Plant Essential Oils

The fumigant toxicities of constituents from three active oils and organophosphorus insecticide dichlorvos are shown in Table 3. Among the test compounds, isopinocamphone and pinocamphone showed 100% fumigant toxicity against *S. oryzae* adults at whole test concentrations, although no mortality was observed at 0.0.375 mg/L air concentration (data not shown here). The insecticidal activities of α-terpineol and terpinen-4-ol were 100% and 88% at 3 mg/L air concentration but were reduced to 18% and 24% at 1.5 mg/L air concentration, respectively. Linalool, camphor, camphene, and sabinene hydrate exhibited 100% fumigant toxicity at 12.5 mg/L air concentration but showed weak activity at 6.5 mg/L air concentration. The fumigant toxicity of terpinolene was 98% at 25 mg/L air concentration, but decreased to 20% at 12.5 mg/L air concentration. Other compounds exhibited moderate or weak fumigant toxicity at 25 mg/L air concentration. Dichlorvos showed 100% fumigant toxicity at whole test concentrations. Kim *et al.* [24] already reported that the LC_50_ value of dichlorvos against rice weevil adults was 0.0081 mg/L air concentration. Insecticidal activities of essential oil constituents against rice weevil are well documented [1,4,5]. Lee *et al.* [5] reported that 1,8-cineole, *p*-cymene, limonene, linalool, α-terpinene, α-terpineol, and terpinen-4-ol exhibited fumigant toxicity against *S. oryzae* adults. However, fumigant toxicity of 1,8-cineole, *p*-cymene, and limonene was not observed in this study. LC_50_ values of camphene and camphor against *S. oryzae* adults were >100 mg/L air concentration in a previous study [4], but the fumigant toxicities of two compounds in this study were stronger than those of a previous study. This difference might be attributed to the use of different strains of *S. oryzae*. Although essential oil and solvent extracts of hyssop have been documented to show antimicrobial, antioxidant, antiviral, cytotoxicity, and α-glucosidase inhibitory activity, the biological activities of pinocamphone and isopinocamphone, main constituents of hyssop oil, have not been well documented [25]. In this study, we isolated two main components from hyssop oil and determined their fumigant toxicities against *S. oryzae* adults for the first time.

The structure-activity relationships of essential oil constituents have been well documented [26,27]. Usually, the insecticidal or nematicidal properties of essential oil constituents with aldehyde, ketone, or alcohol functional groups are stronger than those of monoterpenoids belonging to hydrocarbons. Kim *et al.* [28] also reported that the fumigant toxicities of oil constituents with aldehyde, ketone, and alcohol groups were much stronger than those of hydrocarbons such as α-pinene, β-pinene, myrcene, *p*-cymene, and limonene against rice weevil adults. In this study, similar results were observed. The fumigant toxicities of oil constituents belonging to alcohol or ketone groups were much stronger than those of hydrocarbon terpenoids. However, a different relationship was found in a test with the Asian tiger mosquitoe, *Aedes albopictus*. Seo *et al.* [7] reported that larvicidal activities of hydrocarbon terpenoids were stronger than those of alcohols, aldehydes, and ketones. This result indicated that essential oil constituents might have different modes of action according to the insect species.

### 2.4. Comparative Toxicities of Artificial Blends

**Figure 1:**
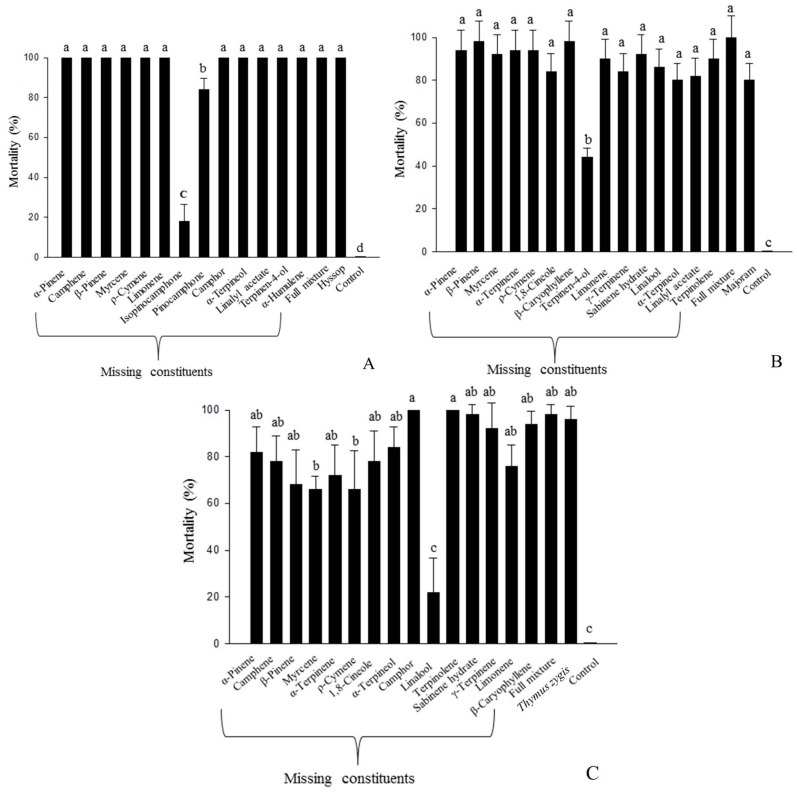
Fumigant toxicities of hyssop (**A**); majoram (**B**); *Thymus zygis* oil (**C**), a full mixture, and selected blends of the constituents in rice weevil adults with 48 h of treatment. The concentration of hyssop, majoram, and *Thymus zygis* oil was 12.5 mg/L air. The concentrations of the full mixture of hyssop, majoram, and *Thymus zygis* oil were 9.86, 9.74, and 10.34 mg/L air, respectively. The concentrations of the other blends were determined by removing each constituent equivalent to the ratio identified in hyssop, majoram, and *Thymus zygis* oil. Mean values corresponding to each treatment with different letters are significantly different from each other (hyssop oil: F_15,64_ = 774.92, *p* < 0.0001; majoram oil: F_17,72_ = 38.125, *p* < 0.0001; *Thymus zygis* oil: F_17,72_ = 37.70, *p* < 0.0001; Scheffé’s test).

The fumigant toxicities of artificial blends against rice weevils are shown in Figure 1. There was no significant difference in the fumigant toxicities between artificial mixtures including all constituents and essential oils (Figure 1). In a component elimination test of hyssop oil, omission of isopinocamphone caused a significant decrease in the fumigant toxicity (F_15,64_ = 774.92, *p* < 0.0001). This result indicated that isopinocamphone was a major contributor to the fumigant toxicity of hyssop oil. Although the decrease in fumigant toxicity was lower compared to that of isopinocamphone, omission of pinocamphone also caused a significant difference in fumigant toxicity (Figure 1A). This result showed that pinocamphone was the second main contributor to the fumigant toxicity of hyssop oil. Omission of other compounds from the full mixture did not cause a significant difference in the fumigant toxicity of the blend. For majoram oil, a component elimination assay showed that the omission of terpinen-4-ol from the full mixture caused a significant decrease in the fumigant toxicity of the blend (F_17,72_ = 38.125, *p* < 0.0001). Omission of other constituents did not cause a significant difference in fumigant toxicity. This result indicated that terpinen-4-ol was the major contributor to fumigant toxicity of majoram oil. Omission of linalool, myrcene, and *p*-cymene from the artificial full mixture of *Thymus zygis* oil components showed a significant difference in the fumigant toxicity of the blend (F_17,72_ = 37.70, *p* < 0.0001). Linalool was the major contributor to the fumigant toxicity of *Thymus zygis* oil, followed by *p*-cymene and myrcene. This result indicated that linalool, *p*-cymene and myrcene act synergistically in terms of fumigant toxicity against rice weevils. A comparative toxicity test of artificial blends showed that there was a major contributor to the fumigant toxicity of active oils, and this information could be very useful for determining the minimum ratio of major contributors. Furthermore, our results also indicated that components of essential oils act synergistically in fumigant toxicity.

## 3. Materials and Methods

### 3.1. Plant Essential Oils and Chemicals

Essential oils of patchouli (*Pogostemon patchouli*), *Salvia stenophylla*, and *Thymus zygis* were purchased from Oshadhi (Bühl/Baden, Germany, www.oshadhi.eu). Hyssop (*Hyssopus officinalis*), litesa (*Litsea cubeba*), lavender (*Lavandula officinalis*), majoram (*Origanum majorana L.*), and sage spanish (*Salvia officinalis*), were purchased from JinArome (Anyang, Korea, www.jinarome.com). *α*-Pinene (purity, 99%), camphene (80%), β-pinene (99%), *p*-cymene (99%), 1,8-cineole (99%), limonene (97%), camphor (99%), and *α*-humulene (96%) were obtained from Sigma-Aldrich (Milwaukee, WI, USA). α-Terpinene (85%), and sabinene hydrate (97%) were purchased from Fluka (Buchs, Switzerland). (−)-α-Phellandrene (65%), terpinolene (85%), (±)-terpinen-4-ol (95%), and *β*-caryophyllene (90%) were obtained from the Tokyo chemical industry (Tokyo, Japan). Acetone was purchased from Merck (Darmstadt, Germany) (99.8%).

### 3.2. Insects

A culture of *S. oryzae* was maintained in the laboratory without exposure to any insecticide. The weevils were reared on unpolished rice in plastic cages (60 × 40 × 40 cm) at 25 ± 1 °C and 60% relative humidity (RH) under a 16:8 h light:dark cycle.

### 3.3. Gas Chromatography

Gas chromatography analysis of hyssop, majoram, and *Thymus zygis* was performed using an Agilent 7890N (Santa Clara, CA, USA) equipped with a flame ionization detector (FID). A 1 μL sample of the essential oil dissolved in hexane (essential oil:hexane = 1:200, *v*/*v*) was injected. DB-1MS (30 m × 0.25 mm i.d., film thickness: 0.25 μm, J & W Scientific, Santa Clara, CA, USA) and HP-INNOWAX columns (30 m × 0.25 mm i.d., film thickness: 0.25 μm, J & W Scientific) were individually used for separation of the essential oil constituents. The oven temperature was programmed as isothermal at 40 °C for 1 min, raised to 250 °C at a rate of 6 °C/min, and held at this temperature for 4 min. Helium was used as the carrier gas, and the flow rate was 1.5 mL/min. The retention indices were obtained in relation to a homologous series of *n*-alkanes (C_7_–C_20_), under the same operating conditions used for the GC-FID analysis. The components were further confirmed by enhancing the integrated area by co-injection with the essential oil and standard samples.

### 3.4. Gas Chromatography-Mass Spectrometry

A gas chromatograph (Agilent 7890A, Santa Clara, CA, USA) and a mass spectrometer (Agilent 5975C MSD) were used for comparing the mass spectra of each peak to those obtained from the NIST MS library using a DB-1MS column (30 m × 0.25 mm i.d., film thickness: 0.25 μm, J & W Scientific). A 1 μL sample of the essential oil dissolved in hexane (essential oil:hexane = 1:200, *v*/*v*) was injected. The oven temperature was the same as that used for the GC-FID analysis. The flow rate of the carrier gas (helium) was 1.0 mL/min. The GC column effluent was introduced directly into the MS source via a transfer line at 250 °C. Ionization was obtained by electron impact (70 eV, source temperature: 230 °C), and the scan range was 41–400 amu.

### 3.5. Isolation of Isopinocamphone and Pinocamphone

**Figure 2:**
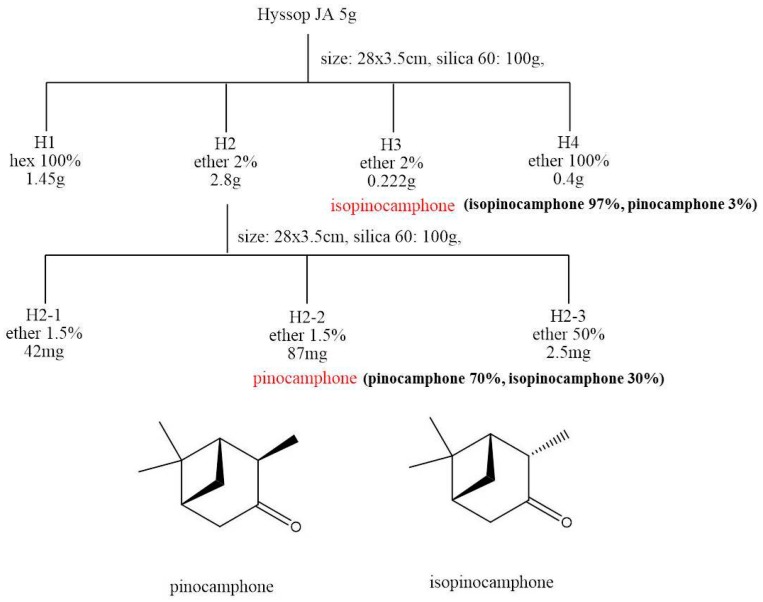
Isolation procedure of isopinocamphone and pinocamphone, and their chemical structures.

To isolate isopinocamphone and pinocamphone, which were not commercially available, we used open column chromatography (Figure 2). Small amounts of hyssop (5 g) oil were subjected to SiO_2_ gel column chromatography (hexane/ether 100/0→0/100). Almost pure iospinocamphone (0.222 g, 97%) was isolated from the diethyl ether 2% fraction. The H2 fraction was further subjected to SiO_2_ gel column chromatography, and pinocamphone (87 mg, mixture of 30% isopinocamphone) was isolated from the ether 1.5% fraction. Purity of pinocamphone and isopinocamphone was determined with gas chromatography. Isolated compounds were identified by gas chromatography-mass spectrometry and used for fumigant and comparative toxicity tests.

### 3.6. Fumigant Toxicity Test

Lamiaceae plant essential oils or their constituents were applied to a paper disc (8 mm, Advantec, Tokyo, Japan) that was placed in the bottom lid of a glass cylinder (diameter, 5 cm; height, 10 cm) with a wire sieve fitted 3.5 cm above the bottom. The lid of the glass cylinder was then sealed with para-film (Pechiney Plastic Packaging Company, Chicago, IL, USA). Ten rice weevil adults (10–20 days old) were placed on the sieve, and this prevented direct contact of the rice weevils with the test oils or compounds. Glass cylinders were kept at 25 ± 1 °C and 60% RH. Adult rice weevils were considered dead if their appendages did not move when prodded with a paintbrush. Cumulative mortality was determined 48 h after treatment. All treatments were replicated five times.

### 3.7. Comparative Toxicity Test

To know the role of each constituent for fumigant toxicity against rice weevils, we made artificial blends made by all available constituents of hyssop, majoram and *Thymus zygis* essential oils. We also made a number of blends that omitted one constituent from the mixture of all constituents (Figure 1). Blends were based on the natural composition of constituents determined by GC-FID (Table 2). The concentrations of the full mixture of hyssop, majoram, and *Thymus zygis* essential oils were 9.86 mg/L, 9.74 mg/L, and 10.34 mg/L air concentration, respectively. The concentrations of other artificial blends were prepared by removing each constituent equivalent to the ratio identified in hyssop, majoram, and *Thymus zygis* essential oils.

### 3.8. Statistical Analysis.

The percentage of mortality for *S. oryzae* adults was transformed to arcsine square-root values prior to analysis of variance (ANOVA). Treatment mean values were compared and separated using Scheffe’s test. Mean (±SE) values of untransformed data have been reported. Statistical analyses were performed using IBM SPSS Statistics 23.0 (2015).

## 4. Conclusions

Of the eight species belonging to Lamiaceae, hyssop (*Hyssopus offcinalis*), majoram (*Origanum majorana*), and *Thymus zygis* essential oils showed strong fumigant toxicity against *S. oryzae* adults. Among identified constituents from active oils, pinocamphone and isopinocamphone showed the strongest fumigant toxicity against *S. oryzae*. The measured toxicity of the artificial blends of the constituents identified in hyssop, majoram, and *Thymus zygis* oils indicated that isopinocamphone, terpine-4-ol, and linalool were major contributors to the fumigant toxicity of the artificial blend, respectively.

## Figures and Tables

**Table 1:** Fumigant toxicities of Lamiaceae plant essential oils against adults of the rice weevil *S. oryzae*.

Plant Essential Oils	Mortality (%, Mean ± S.E., N ^a^ = 50)			
	25 ^b^	12.5	6.5	3.0
Hyssop	100a ^c^	100a	100a	6 ± 4.9a
Lavender	68 ± 9.8b	28 ± 9.8c	16 ± 10.2c	-
Litesa	70 ± 8.9b	8 ± 4.0d	-	-
Majoram	100a	80 ± 12.6b	14 ± 8.0c	-
Patchouli	0d	- ^d^	-	-
*Sage spanish*	24 ± 8.0c	-	-	-
*Salvia stenophylla*	0d	-	-	-
*Thymus zygis*	100a	96 ± 4.9a	74 ± 12.0b	6 ± 8.0a
Control	0d	0d	0c	0a
	F_8,36_ = 307.20 *p* < 0.0001	F_5,24_ = 187.15 *p* < 0.0001	F_4,20_ = 121.87 *p* < 0.0001	F_2,12_ = 1.636 *p* = 0.235

^a^: Number of insects tested. ^b^: mg/L air concentration; ^c^: Means within a column followed by the same letters are not significantly different (Scheffe’s test); ^d^: Not tested.

**Table 2:** Chemical analysis of active essential oils.

No.	Compound	Retention Index		Composition Ratio (%)		
		DB-1MS/Lit	HP-Innowax	Hyssop	Majoram	*Thymus zygis*
1	α-Pinene	929/940 ^a^	1020	0.65	0.44	3.80
2	Camphene	941/950 ^a^	1063	0.11	-	1.12
3	β-Pinene	968/975 ^a^	1107	11.67	1.18	0.34
4	Myrcene	982/981 ^a^	1165	1.32	1.02	5.88
6	*α*-Terpinene	1007/1011 ^a^	1180	- ^d^	5.40	1.45
7	*p*-Cymene	1011/1014 ^a^	1273	0.11	6.37	4.14
8	1,8-Cineole	1018/1026 ^a^	1208	-	1.12	0.61
9	Limonene	1020/1018 ^b^	1199	0.92	3.22	2.77
10	γ-Terpinene	1048/1050 ^a^	1247	-	12.92	3.13
11	Sabinene hydrate	1051/1056 ^a^	1465	-	2.04	5.35
12	Terpinolene	1077/1081 ^a^	1284	-	1.07	0.84
13	Linalool	1084/1083 ^a^	1560	-	15.32	40.14
14	Camphor	1117/1128 ^a^	1520	13.25	-	0.36
15	Pinocamphone	1135/1142 ^a^	1520	13.25	-	-
16	Isopinocamphone	1145/1157 ^a^	1550	35.68	-	-
17	Terpinen-4-ol	1160/1166 ^a^	1610	0.17	22.96	8.83
18	α-Terpineol	1171/1178 ^a^	1705	0.24	2.50	2.30
19	Linalyl acetate	1240/1240 ^a^	1560	1.40	0.85	0.62
20	β-Caryophyllene	1414/1429 ^a^	1599	-	1.58	1.07
21	α-Humulene	1447/1450 ^c^	1672	0.16	-	-
	Sum			78.93	77.99	82.75

^a^: Salehi *et al.* [21]; ^b^: Muselli *et al.* [22]; ^c^: Sefidkon [23]; ^d^: Not detected.

**Table 3:** Fumigant toxicities of constituents identified from active plant essential oils against adults of the rice weevil *S. oryzae*.

Compounds	Mortality (%, Mean ± S.E., N ^a^ = 50)					
	25 ^b^	12.5	6.5	3.0	1.5	0.75
α-Pinene	0c ^c^	- ^d^	-	-	-	-
Camphene	100a	100a	10 ± 6.3cd	-	-	-
β-Pinene	0c	-	-	-	-	-
Myrcene	0c	-	-	-	-	-
α-Terpinene	52 ± 7.5b	0c	-	-	-	-
*p*-Cymene	0c	-	-	-	-	-
1,8-Cineole	0c	-	-	-	-	-
Limonene	0c	-	-	-	-	-
γ-Terpinene	0c	-	-	-	-	-
Sabinene hydrate	100 a	100a	26 ± 13.6c	4 ± 4.9c	-	-
Terpinolene	98 ± 4.0a	20 ± 6.3b	0d	-	-	-
Linalool	100a	100a	74 ± 12.0b	46 ± 10.2b	12 ± 7.5bc	-
Pinocamphone	100a	100a	100a	100a	100a	100a
Isopinocamphone	100a	100a	100a	100a	100a	100a
Camphor	100a	100a	22 ± 7.5c	0d	-	-
α-Terpineol	100a	100a	100a	100a	18 ± 13.3bc	6 ± 4.9b
Linalyl acetate	0c	-	-	-	-	-
Terpinen-4-ol	100a	100a	100a	88 ± 16.0a	24 ± 13.6b	4 ± 4.9b
β-Caryophyllene	0c	-	-	-	-	-
α-Humulene	0c	-	-	-	-	-
Dichlorvos	100a	100a	100a	100a	100a	100a
Control	0c	0c	0d	0c	0c	0b
	F_21,88_ = 3037.76 *p* < 0.0001	F_11,48_ = 2167.27 *p* < 0.0001	F_10,44_ = 211.95 *p* < 0.0001	F_8,36_ = 207.60 *p* < 0.0001	F_6,28_ = 147.42 *p* < 0.0001	F_5,24_ = 1403.53 *p* < 0.0001

^a^: Number of insects tested; ^b^: mg/L air concentration; ^c^: Means within a column followed by the same letters are not significantly different (Scheffe’s test); ^d^: Not tested.
